# 

**Published:** 1948

**Authors:** 


### CONTENTS

| | Page |
|---|---|
| **Bronchial Carcinoma.** By W. W. D. THOMSON, D.L., M.D., B.Sc., F.R.C.P. | 69 |
| **Some Points in the Treatment of Diabetes.** By G. A. SMART, M.D., B.Sc., M.R.C.P. | 76 |
| **Blue Sclerotics, Fragilitas Ossium and Deafness: Report of a Family.** By E. WATSON-WILLIAMS, *M.C.*, M.D., Ch.M., F.R.C.S. | 82 |
| **Thiouracil in Thyrotoxicosis.** | 87 |
| **Reviews of Books** | 88 |
| **Meetings of Societies** | 90 |
| **The Medical Library of the University of Bristol** | 93 |
| **Publications Received** | 95 |
| **Local Medical Notes** | 96 |

